# Diagnostic Challenges in Acute Infantile Epididymitis: A Case Report

**DOI:** 10.7759/cureus.82196

**Published:** 2025-04-13

**Authors:** Manabu Watari, Shohei Yoshimura, Hiroyuki Nagao, Kengo Hattori, Yo Okizuka

**Affiliations:** 1 Department of Pediatrics, Takatsuki General Hospital, Takatsuki, JPN; 2 Department of Pediatric Surgery, Takatsuki General Hospital, Takatsuki, JPN; 3 Department of Pediatric Surgery, Hyogo Prefectural Kobe Children's Hospital, Kobe, JPN

**Keywords:** acute epididymitis, acute scrotum, c-reactive protein, emergency surgery, strangulated inguinal hernia, testicular torsion

## Abstract

Acute epididymitis is a rare yet significant cause of acute scrotum in infants, which presents diagnostic challenges owing to its similarity to other conditions requiring emergency surgery, such as strangulated inguinal hernia and testicular torsion. This report describes two cases of acute epididymitis in infants, emphasizing the importance of differential diagnosis in the emergency department. In case 1, a six-month-old male infant with swelling and erythema extending from the right groin to the scrotum was initially suspected of having a strangulated inguinal hernia. However, scrotal ultrasonography and urinalysis confirmed an acute epididymitis, and the patient responded well to antibiotics. In case 2, a three-month-old male infant with scrotal erythema and swelling required surgical exploration to rule out testicular torsion and was ultimately diagnosed with acute epididymitis. The patient was cured with antibiotics without relapsing. This report underscores the role of clinical evaluation, scrotal ultrasound, and laboratory tests such as urinalysis and serum C-reactive protein levels in diagnosing epididymitis and differentiating it from other acute scrotal conditions. However, surgical exploration is a useful diagnostic tool for acute infantile epididymitis. Early and accurate diagnosis of acute epididymitis using these examinations is crucial for preventing long-term complications.

## Introduction

Acute epididymitis is a common inflammatory condition of the epididymis that presents with scrotal pain, swelling, erythema, and occasionally fever and is a leading cause of acute scrotum in pediatric patients presenting to the emergency department. Nevertheless, it is relatively uncommon to diagnose it in infants, with only a small percentage of all pediatric patients being diagnosed with this condition [1]. Furthermore, in this age group, it is difficult to explain their symptoms, and other diseases that require emergency surgery, such as strangulated inguinal hernia and testicular torsion, must be considered [2].

Scrotal ultrasound is an essential diagnostic tool for differentiating acute epididymitis, inguinal hernia, and testicular torsion. Acute epididymitis is characterized by epididymal swelling and increased blood flow to the affected epididymis on scrotal ultrasound [3]. In contrast, inguinal hernia typically presents with bowel herniation within the processus vaginalis [4], and testicular torsion is characterized by inadequate blood flow to the affected testis and twisting of the spermatic cord, known as the "whirlpool sign" on scrotal ultrasound [5,6].

It is challenging for pediatricians to diagnose acute epididymitis in infants in the emergency department because reaching a definitive diagnosis based on scrotal ultrasound requires sufficient experience and technical skill, and misdiagnosis of epididymitis may lead to unnecessary surgical exploration [7]. For acute epididymitis, antibiotic therapy with cephalosporins is generally administered for 7 to 14 days for cure; however, delayed diagnosis of acute epididymitis may lead to complications such as testicular infarction, abscess formation, and long-term infertility [8,9]. Herein, we describe two infants with acute epididymitis that required differentiation from other conditions requiring emergency surgery, such as a strangulated inguinal hernia and testicular torsion.

## Case presentation

Case 1

**Figure 1:**
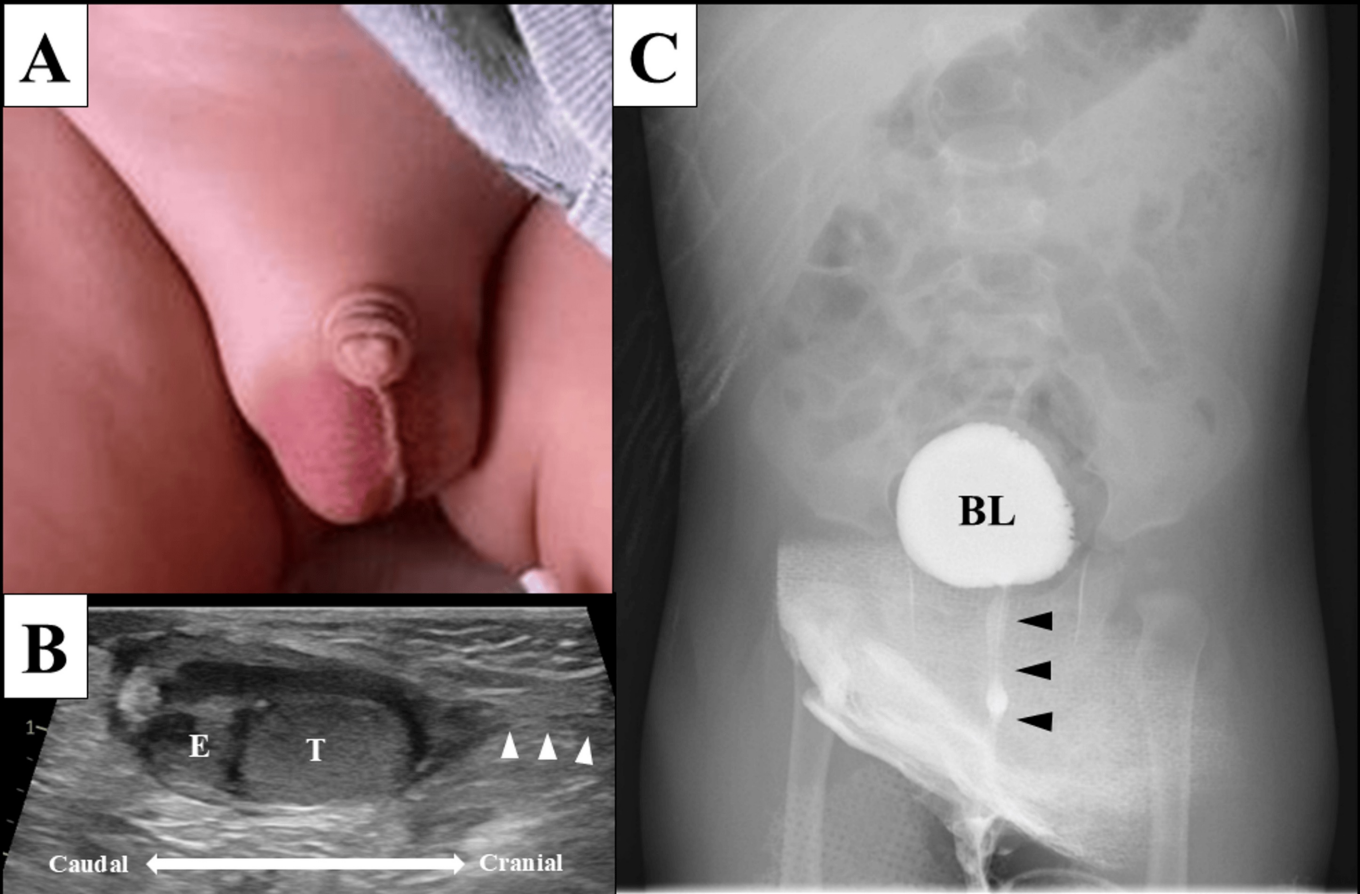
Physical and imaging findings in Case 1 A: Swelling and erythema from the right groin to the scrotum, and suspicion of an incarcerated inguinal hernia. B: Scrotal ultrasound image showing enlargement of the inferior epididymis and no patent processus vaginalis (white arrowheads). C: Voiding cystourethrography showing no vesicoureteral reflux or lower urinary tract abnormalities (black arrowheads). BL, bladder; E, epididymis; T, testis

A previously healthy six-month-old male infant was referred to the pediatric emergency department with a suspected strangulated inguinal hernia. The patient presented with two days of swelling and erythema extending from the right groin to the scrotum (Figure 1A), accompanied by irritability and a fever of 38.4℃. The serum C-reactive protein (CRP) level was elevated at 1.93 mg/dL (normal range: <0.30 mg/dL), and the white blood cell count increased to 22,000/µL (normal range: 4,400-19,100/µL). Scrotal ultrasonography showed enlargement of the epididymis on the inferior side of the testis and no patent processus vaginalis, suggesting an acute epididymitis rather than a strangulated inguinal hernia (Figure 1B). Urinalysis revealed pyuria with a white blood cell count of 65/µL, supporting acute epididymitis. Intravenous cefmetazole 100 mg/kg/day was administered for four days, and fever and right scrotal swelling gradually resolved. Finally, the blood culture result was negative; however, the urine culture result was positive for 1 × 10^5^ *Escherichia coli*, confirming the diagnosis of infantile bacterial epididymitis. On the fourth day of hospitalization, urological ultrasound indicated no urological anomalies and showed a testicular size of 15 × 10 × 9 mm on the right and 13 × 10 × 7 mm on the left. The patient was discharged on the sixth day of hospitalization and continued oral cefaclor administration for an additional 10 days, completing a total of 14 days of antibiotic therapy. Voiding cystourethrography (VCUG), performed on post-discharge day 28, revealed no urinary tract malformations (Figure 1C). The infant has had no recurrence of epididymitis for a year.

Case 2

**Figure 2:**
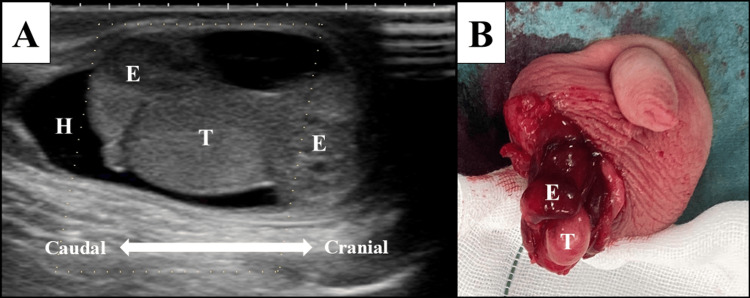
Imaging and operative findings in Case 2 A: Scrotal ultrasound image showing enlargement of the entire epididymis, hydrocele, and lack of blood flow within the testis, suggesting epididymitis or testicular torsion. B: Surgical findings showing inflammatory enlargement of the epididymis with no testicular torsion, leading to a definitive diagnosis of epididymitis. E, epididymis; H, hydrocele; T, testis

A previously healthy three-month-old male infant presented to the pediatric emergency department with erythema and swelling of the right scrotum, which developed on the day of presentation, along with irritability and a fever of 37.7℃. The serum CRP level was elevated at 1.66 mg/dL (normal range: <0.30 mg/dL), and urinalysis revealed pyuria with a white blood cell count of >500/µL (normal range: 4,400-19,100/µL). Scrotal ultrasonography revealed a right-sided hydrocele, enlargement of the right epididymis, and indistinct blood flow in the right testis (Figure 2A). Testicular torsion could not be ruled out, and emergency surgical exploration was performed. Intraoperative findings revealed no evidence of testicular torsion, but inflammatory enlargement of the right epididymis, leading to a diagnosis of acute epididymitis (Figure 2B). Intravenous cefmetazole 100 mg/kg/day was administered for three days, and the symptoms gradually resolved. Finally, both the blood and urinary cultures were negative.

The patient was discharged on the fourth day of hospitalization and continued oral cefaclor administration for an additional four days, completing a total of seven days of antibiotic therapy. Urological ultrasound performed on the ninth day post-discharge showed a right testicular size of 14 × 9 mm and a left testicular size of 14 × 5 mm. On the 44th day post-discharge, a follow-up urological ultrasound showed a right testicular size of 15 × 10 mm and left testicular size of 14 × 8 mm. According to these findings, there was no evidence of testicular atrophy or abscess formation. The infant has had no recurrence of epididymitis for a year.

Table 1 summarizes the key findings in both cases.

**Table 1:** Summary and comparison of clinical courses in two cases of infantile acute epididymitis Comparative summary of clinical presentation, diagnostic evaluation, management, and outcomes in two infantile cases of acute epididymitis. Despite initial concern for surgical emergencies, both cases were ultimately diagnosed with epididymitis and treated successfully with antibiotics. CAKUT: Congenital anomalies of the kidney and urinary tract

Feature	Case 1	Case 2
Ultrasound findings	Enlarged epididymis with increased blood flow; normal testis	Equivocal testicular blood flow; inflamed epididymis
Urinalysis	Pyuria	Pyuria
CRP level	Mildly elevated	Elevated
Surgical exploration	Not performed	Performed; no torsion found
Final diagnosis	Acute epididymitis	Acute epididymitis
Antibiotic used	Cefmetazole	Cefmetazole
Response to treatment	Rapid improvement	Rapid improvement
CAKUT evaluation	Renal and bladder ultrasound: normal	Renal and bladder ultrasound: normal
Outcome	Full recovery without recurrence	Full recovery without recurrence

## Discussion

In this case report, we discuss the cases of two infants with acute epididymitis. In the first case, acute epididymitis was diagnosed based on pyuria, and an inflamed and enlarged epididymis was observed on scrotal ultrasound despite the initial suspicion of a strangulated inguinal hernia. However, in the other case, surgical exploration was necessary to confirm the diagnosis of acute epididymitis and rule out testicular torsion. Infants with acute epididymitis, strangulated inguinal hernia, and testicular torsion often present to the emergency department with symptoms of the acute scrotum, making it challenging for pediatricians to make a definite diagnosis. In addition, it is difficult to make an accurate differential diagnosis based on physical findings, such as scrotal pain, swelling, erythema, fever, and irritability [7,10].

The standard diagnostic tool for acute epididymitis is scrotal ultrasound. Elevated serum CRP and pyuria on urinalysis are accessory diagnostic tools for acute epididymitis [11]. Liu et al. revealed that an elevated serum CRP level was a more accurate and reliable tool than urinalysis for predicting acute epididymitis in pediatric patients [11]. Joo et al. showed that acute epididymitis cannot be ruled out from urinary findings because only 3.9% and 1.3% of children with acute epididymitis had positive findings for pyuria and urinary culture, respectively [12]. Although both cases in this report were associated with elevated serum CRP levels and pyuria, it was difficult to make a definitive diagnosis of acute epididymitis in the second case based on ultrasound findings, indicating the limitations of scrotal ultrasound in infants. Doppler ultrasound remains the gold standard for distinguishing acute epididymitis from testicular torsion because testicular torsion typically results in reduced or absent blood flow to the affected testis [7,10]. However, 44% of infants who underwent surgical exploration for acute scrotum were ultimately diagnosed with acute epididymitis based on operative findings [13]. Scrotal ultrasound, including color Doppler imaging, is a valuable tool but may have limited sensitivity in detecting testicular blood flow in young infants due to the small size of vessels and technical difficulties. Pediatricians should consult pediatric surgeons or urologists to facilitate surgical exploration and diagnosis in cases that are difficult to differentiate from other acute scrotal diseases.

Infantile epididymitis often involves urinary tract anomalies [1,14,15]. Delayed or incorrect diagnosis of epididymitis can lead to severe complications, including testicular infarction, abscess formation, and long-term infertility [8,9]. Therefore, follow-up imaging and evaluation of urinary tract malformations are essential to identify potential predisposing factors for recurrence, including ectopic ureteral opening to the seminal vesicle, posterior urethral valve, and vesicourinary reflux [10,14,15]. The recommended imaging techniques include urologic ultrasound and VCUG, which help identify potential structural abnormalities such as congenital anomalies of the kidney and urinary tract (CAKUT). These conditions can predispose infants to recurrent urinary tract infections (UTIs), which may further complicate the clinical course of epididymitis [10,14]. Merlini et al. revealed that in the group of 11 infants, seven patients had UTIs (63%), eight had urinary tract anomalies (73%), and urinary tract malformations and UTIs were simultaneously present in six infants (55%), suggesting a high prevalence of such anomalies in cases of acute epididymitis in infants [14].

Both infants reported herein underwent comprehensive urinary tract screening following the diagnosis of epididymitis. The first infant had normal findings on urological ultrasound and VCUG, with no underlying CAKUT. This approach confirmed the absence of structural abnormalities, and the patient did not experience epididymitis recurrence. Similarly, the second patient, who initially required surgical exploration to rule out testicular torsion, was confirmed to have acute epididymitis based on intraoperative findings. Postoperative imaging, including follow-up urological ultrasound, revealed no underlying urinary tract malformations, and there was no recurrence of the condition. Table 1 provides a side-by-side comparison of clinical presentation, imaging findings, treatment, and outcomes in the two cases, aiding in future diagnostic consideration. These findings underscore the importance of considering CAKUT in the differential diagnosis of pediatric epididymitis. Pediatricians should be vigilant when performing standard urological imaging, including ultrasonography and VCUG, to assess possible structural abnormalities, particularly in cases of recurrent epididymitis or urinary tract infections. The choice of empirical antibiotic therapy in infants should consider the most likely causative organisms. Gram-negative bacteria, particularly *Escherichia coli*, are the predominant pathogens in infantile epididymitis, especially in those with urinary tract anomalies [14]. Thus, in both our cases, second-generation cephalosporins were selected. In addition, although imaging is crucial in the diagnosis of acute epididymitis, surgical exploration remains an important step when testicular torsion cannot be ruled out through clinical and imaging evaluations because timely intervention is essential to prevent complications such as testicular infarction or long-term fertility [16].

Finally, this case report highlights the importance of considering acute epididymitis in the differential diagnosis of acute scrotum in infants, even in the presence of features suggestive of other conditions, such as strangulated inguinal hernia and testicular torsion. Pediatricians should make an effort to correctly diagnose acute epididymitis based on laboratory and ultrasound findings to prevent unnecessary surgical exploration. Early diagnosis and intervention are crucial in preventing long-term complications of acute epididymitis. Therefore, surgical exploration, while invasive, plays a critical role in cases where testicular torsion cannot be excluded based on clinical and imaging findings.

## Conclusions

Accurate diagnoses of infantile epididymitis through laboratory investigations and scrotal ultrasounds by pediatricians are crucial; however, surgical exploration is a useful diagnostic tool for acute infantile epididymitis. These examinations play a critical role in distinguishing acute epididymitis from other diseases that present with an acute scrotum, and appropriate therapeutic interventions are effective in preventing long-term complications of acute epididymitis. Furthermore, comprehensive evaluation of the urinary tract is essential to identify any underlying anomalies that may predispose to recurrent epididymitis or urinary tract infections.
